# Multicenter Postimplementation Assessment of the Positive Predictive Value of SARS-CoV-2 Antigen-Based Point-of-Care Tests Used for Screening of Asymptomatic Continuing Care Staff

**DOI:** 10.1128/JCM.01411-21

**Published:** 2021-10-19

**Authors:** Jamil N. Kanji, Dustin T. Proctor, William Stokes, Byron M. Berenger, James Silvius, Graham Tipples, A. Mark Joffe, Allison A. Venner

**Affiliations:** a Division of Infectious Diseases, Department of Medicine, University of Calgary School of Medicine, Calgary Alberta, Canada; b Public Health Laboratory, Alberta Precision Laboratories, Alberta, Canada; c Department of Laboratory Medicine and Pathology, Faculty of Medicine and Dentistry, University of Alberta, Edmonton, Alberta, Canada; d Department of Pathology and Laboratory Medicine, University of Calgary School of Medicine, Calgary, Alberta, Canada; e Clinical Biochemistry, Alberta Precision Laboratories, Diagnostic and Scientific Center, Calgary, Alberta, Canada; f Division of Infectious Diseases, Department of Medicine, University of Alberta, Edmonton, Alberta, Canada; g Division of Geriatric Medicine, Cumming School of Medicine, Calgary, Alberta, Canada; h Li Ka Shing Institute of Virology, University of Alberta, Edmonton, Alberta, Canada; i Department of Medical Microbiology and Immunology, Faculty of Medicine and Dentistry, University of Alberta, Edmonton, Alberta, Canada; j Alberta Health Services, Edmonton, Alberta, Canada; Cepheid

**Keywords:** point of care, SARS-CoV-2 antigen, positive predictive value, sensitivity

## Abstract

Frequent screening of severe acute respiratory syndrome coronavirus 2 (SARS-CoV-2) among asymptomatic populations using antigen-based point-of-care tests (APOCTs) is occurring globally with limited clinical performance data. The positive predictive value (PPV) of two APOCTs used in the asymptomatic screening of SARS-CoV-2 among health care workers (HCWs) at continuing care (CC) sites across AB, Canada, was evaluated. Between 22 February and 2 May 2021, CC sites implemented SARS-CoV-2 voluntary screening of their asymptomatic HCWs. On-site testing with Abbott Panbio or BD Veritor occurred on a weekly or twice-weekly basis. Positive APOCTs were confirmed with a real-time reverse transcriptase PCR (rRT-PCR) reference method. A total of 71,847 APOCTs (17,689 Veritor and 54,158 Panbio) were performed among 369 CC sites. Eighty-seven (0.12%) APOCTs were positive, of which 39 (0.05%) were confirmed as true positives using rRT-PCR. Use of the Veritor and Panbio resulted in 76.6% and 30.0% false-positive detection, respectively (*P* < 0.001). This corresponded to PPVs of 23.4 and 70.0% for the Veritor and Panbio, respectively. Frequent screening of SARS-CoV-2 among asymptomatic HCWs in CC, using APOCTs, resulted in a very low detection rate and a high rate of detection of false positives. Careful assessment of the risks versus benefits of APOCT programs and the prevalence of infection in this population needs to be thoroughly considered before implementation.

## INTRODUCTION

Point-of-care testing (POCT), and specifically antigen-based point-of-care tests (APOCTs), represent an important public health measure in managing severe acute respiratory syndrome coronavirus 2 (SARS-CoV-2) transmissions when used appropriately and with acknowledgment of their shortcomings. The APOCT is particularly useful in identifying and isolating SARS-CoV-2-positive cases in symptomatic individuals in high-risk settings, such as congregate housing and care facilities, or in areas where longer turnaround times (TATs) of more sensitive nucleic acid testing may compromise containment of transmission. Governments around the world have been quick to support the wide-scale implementation of APOCT programs (1).

Rapid and large-scale deployment of APOCTs has been promoted despite limited data regarding the analytical and clinical performance of these devices in the various settings in which they have been used. POCT programs that include APOCTs have been criticized for the poor sensitivity of these tests compared to PCR tests, particularly in asymptomatic individuals (2). However, there has been intense interest in testing asymptomatic individuals from a public health perspective, as some estimate that 30 to 40% of infectious individuals are asymptomatic. Rapid identification and isolation of these individuals are viewed as highly effective in breaking chains of transmission (1, 3–5). However, there is concern that APOCTs may demonstrate poor specificity when used for the surveillance of asymptomatic individuals, with reports citing false-positive rates of up to 60% (6, 7).

Routine serial testing of asymptomatic staff in continuing care facilities using APOCTs has been implemented as a purportedly important public health measure for early case identification and prevention of SARS-CoV-2 outbreaks among vulnerable residents (1, 3–5). While APOCTs may represent an important tool in limiting transmission in congregate settings, the negative impacts of a false-positive result for staff and the site need to be considered, and further data on the clinical performance of APOCTs in continuing care facilities are required.

Our aim was to evaluate the positive predictive values (PPVs) of two APOCTs (Abbott Panbio and BD Veritor) in a large-scale multicenter implementation of asymptomatic SARS-CoV-2 testing of health care workers (HCWs) in continuing care facilities.

## MATERIALS AND METHODS

### Setting.

Asymptomatic SARS-CoV-2 testing of HCWs in continuing care facilities provincewide was implemented as POCT as part of the provincial pandemic response in the province of Alberta, Canada (population of 4.4 million) (8). A retrospective review of test results was completed for testing completed from 22 February 2021 to 2 May 2021 inclusive. Facilities were assigned (based on availability, oversight, and preference) to use either the Panbio COVID-19 antigen rapid test (Abbott Laboratories, Chicago, IL, USA) or the Veritor COVID-19 rapid antigen test (Becton Dickson and Company, Franklin Lakes, NJ, USA), herein referred to as APOCTs.

Early in the pandemic, the provincial health authority had instituted a continuous masking policy for all continuing care facilities, where all staff were required to continuously wear a medical mask while at work, to be changed should the mask become soiled or wet (9). Adjunctive eye protection (face shield or goggles) was advised during all episodes of patient care, or continuously if there was a SARS-CoV-2 outbreak declared at the site (10). HCWs at all facilities were required to complete a “fit for work” symptom questionnaire twice per shift and immediately leave their shift after informing their manager and arrange COVID-19 testing should they develop symptoms or screen positive (11).

### Asymptomatic testing.

Each site received training for a limited number of on-site health care workers to conduct voluntary asymptomatic staff testing of site staff on a weekly basis. Training, operating procedures, and medical/operational oversight were provided by Alberta Precision Laboratories (APL) POCT (for sites run by Alberta Health Services [AHS]). Sites external to AHS had vendor-driven training, and medical/operational oversight was provided by the site. Staff conducting testing wore personal protective equipment (PPE) consisting of eye protection, a surgical mask, gloves, and a splash-resistant gown. Sampling was done using the nasal swab provided in each test kit, with testing conducted as per the manufacturer’s guidelines (12, 13). While awaiting results, staff were permitted to continue working. Staff who were symptomatic were not permitted to receive POCT asymptomatic testing and were directed to access COVID-19 testing through public health test centers. At the discretion of individual facilities, staff testing could be increased to twice weekly for non-outbreak-related staff at a site where a COVID-19 outbreak had been declared (staff considered linked to the outbreak were not eligible for asymptomatic testing).

Staff receiving positive APOCT results were required to immediately isolate and obtain a confirmatory throat or nasopharyngeal swab test for SARS-CoV-2 with a real-time reverse transcriptase PCR (rRT-PCR) result at an approved public health test center. rRT-PCR testing was carried out at the provincial Public Health Laboratory (ProvLab) using a validated laboratory-developed assay (14) or a Health Canada-approved test at an accredited laboratory. Confirmatory rRT-PCR testing was not conducted on negative SARS-CoV-2 APOCT results. Individuals with invalid APOCT results were referred for rRT-PCR evaluation at an approved public health test center, similar to those with a positive test. APOCT test results were not recorded in the medical chart of HCWs. Any positive APOCT results were considered presumptive positive and were not included in total provincial COVID-19 case counts, using instead the confirmatory rRT-PCR results. All HCWs with a positive APOCT result were able to obtain a follow-up rRT-PCR test.

### Data extraction and analysis.

Data recording, organization, and provision of results were provided by Seniors Health and Continuing Care (AHS). Confirmatory rRT-PCR testing results were extracted from the ProvLab laboratory information system. Data were tabulated in Microsoft Excel. Proportional comparisons were done using a two-sample proportions Z-test. Continuous variables were compared using Mann-Whitney tests, while categorical variables were compared using chi-square or Fisher’s exact tests. Significance was set at *P* < 0.05. Confidence intervals (CIs) were calculated using Wilson’s method. Statistical analysis was conducted using StatPlus (AnalystSoft, Inc., Alexandria, VA, USA).

### Ethics approval.

Presentation of the data contained in this report has been approved by the Human Research Ethics Board at the University of Alberta (study identifier Pro00110831).

### Data availability.

The data sets used and/or analyzed during the current study are available from the corresponding author on reasonable request.

## RESULTS

Over the 10-week period, 369/466 (79.18%) provincial continuing care sites participated in the asymptomatic screening program (Table 1). During the study period, 71,847 APOCTs were conducted (mean, 7,184.7 tests per week; range, 615 to 11,648 [17,689 on the Veritor and 54,158 on the Abbott Panbio]) (Table 2). A total of 87 (0.12%) APOCTs were positive, of which 39 (44.83%) were confirmed to be true positives using rRT-PCR (time duration between APOCT and rRT-PCR confirmation not available). The APOCT positivity rate did not differ significantly across the 10-week period (*P* = 0.35) (Table 1).

**TABLE 1:** Weekly APOCT results for asymptomatic staff at continuing care sites across Alberta, Canada

Week^*a*^	No. of reporting sites	No. of tests performed	No. (%) of APOCTs		
			Positive^*b*^	Invalid	Confirmed by rRT-PCR^*c*^
22–28 Feb 2021	11	615	1 (0.16)	4 (0.65)	0
1–7 Mar 2021	115	2,446	0	4 (0.16)	0
8–14 Mar 2021	188	4,374	3 (0.07)	16 (0.37)	0
15–21 Mar 2021	309	8,680	8 (0.09)	29 (0.33)	1 (12.50)
22–28 Mar 2021	293	7,603	6 (0.08)	4 (0.05)	1 (16.67)
29 Mar to 4 Apr 2021	311	8,029	9 (0.11)	13 (0.16)	5 (55.56)
5–11 Apr 2021	317	9,599	12 (0.13)	3 (0.03)	8 (66.67)
12–18 Apr 2021	325	9,312	15 (0.16)	4 (0.04)	6 (40.00)
19–25 Apr 2021	321	9,541	17 (0.18)	6 (0.06)	10 (58.82)
26 Apr to 2 May 2021	369	11,648	16 (0.14)	4 (0.03)	8 (50.00)

Total	369	71,847	87 (0.12)	87 (0.12)	39 (44.83)

*^a^*Feb, February; Mar, March; Apr, April.

b Change in APOCT positivity across the 10 weeks is not significant (Pearson’s chi-square test, *P* = 0.35).

c rRT-PCR, real-time reverse transcriptase PCR.

**TABLE 2:** APOCT and rRT-PCR confirmatory results for asymptomatic continuing care health care workers in AB, Canada

Parameter	Result for:		Total (%)
	BD Veritor	Panbio	
No. (%) of APOCTs			
Total	17,689	54,158	71,847
Positive	47 (0.27)	40 (0.07)	87 (0.12)
Negative	NA^*a*^	NA	71,673 (99.76)
Invalid	NA	NA	87 (0.12)
Positive as confirmed by rRT-PCR	11 (0.06)	28 (0.05)	39 (0.05)

No. (%) of false positives	36/47 (76.60)^*b*^	12/40 (30.00)^*b*^	48/87 (55.17)
Positive predictive value (%)	11/47 (23.40)^*b*^	28/40 (70.00)^*b*^	39/87 (44.83)

a NA, not available.

b For comparison between BD Veritor and Abbott Panbio, *P* < 0.0001.

Compared to the Panbio, the false detection rate was significantly higher using the Veritor (76.6% [95% CI, 62.8 to 86.4%] versus 30.0% [95% CI, 18.1 to 45.4%]; *P* < 0.0001) (Table 2). The PPV was significantly higher with the Panbio test (70% [95% CI, 54.6 to 81.9%] versus 23.4% [95% CI 13.6 to 37.2%]; *P* < 0.0001).

During this time, a total of 66,338 cases of COVID-19 were diagnosed in Alberta (prevalence of 1.5%), with an average rRT-PCR positivity rate (for all specimens tested) of 7.2% (median, 6.3%; range, 3.8 to 13.6%) (15). During the study period, a total of 32 outbreaks were declared in continuing care facilities across the province (one in March 2021, 29 in April 2021, and two in May 2021). HCWs in continuing care facilities were offered mRNA COVID-19 vaccines starting 16 December 2020 alongside the residents in these facilities (with the dosing between first doses being 4 and 6 weeks for residents and HCWs, respectively). The proportions of all HCWs working in Alberta continuing care facilities who had received one dose of COVID-19 vaccine as of 1 February and 2 May 2021 were 1.1 and 69.9%, respectively. The proportions having received two doses of vaccine by these dates were 16.0 and 83.6%, respectively. The demographics and proportions of vaccinated HCWs tested were not available, and neither were the follow-up rRT-PCR testing results of 87 individuals with invalid APOCT results.

## DISCUSSION

Despite great emphasis on the use of APOCTs as a tool to combat the COVID-19 pandemic, expansion of APOCTs for asymptomatic testing has been met with hesitation due to their limited PPV, especially in settings of low disease prevalence (16). This study confirms and quantitates these concerns, demonstrating in a large-scale implementation of asymptomatic screening in continuing care HCWs, that APOCTs have a low overall PPV (44.83%) and a high proportion of false positives (55.17%).

Similar performance concerns related to APOCTs have been raised by a number of studies. A recent Cochrane Database systematic review reported the overall sensitivity (Sn) and specificity (Sp) of APOCTs in symptomatic disease to be 72.0 and 99.5% (27 studies combined) and those in asymptomatic disease to be 58.1 and 98.9%, respectively (12 studies combined) (2). Application of this to the provincial prevalence of disease we observed during the study period (1.5%) indicates our findings are consistent, with an expected PPV for APOCTs around 44.6%. Several studies have reported a PPV as low as 33.3% (17, 18).

Many reports of asymptomatic SARS-CoV-2 testing campaigns using either APOCTs or rRT-PCR tests have demonstrated low test positivity rates, suggesting high-volume testing of asymptomatic individuals is of low yield (19–22). Another Canadian study evaluating universal APOCT screening of HCWs in continuing care also found a similar APOCT positivity rate of 0.16% (23). The prevalence of positive results in our cohort was similar at 0.12%; however, the true prevalence of disease in our cohort is much less if true positives are accounted for (0.05%). Therefore, when considering the true-positivity rate, the yield of asymptomatic screening programs is poor.

The Veritor displayed both a lower PPV and a higher proportion of false-positive tests than the Panbio. Both tests were initially authorized by the FDA and Health Canada for the diagnosis of COVID-19 within the first 5 (Veritor) to 7 (Panbio) days of COVID-19 symptom onset (16). Our findings are consistent with the literature, demonstrating PPVs for the Veritor in asymptomatic individuals ranging from 24.9 to 50.1% (with disease prevalence of 0.5 to 1.5%) (24) and those for the Panbio to be 28.0 to 54.0%, using prevalence estimates of 0.5 to 1.5% (24–26). Thus, the Panbio would be preferred over the Veritor for asymptomatic screening of COVID-19.

The impact of a false-positive result can lead to considerable burden and distress on both individuals and the health care system. False-positive test results in HCWs and others can lead to loss of income due to the need to isolate, collateral effects on close contacts, and also psychological damage due to misdiagnosis, stigma, and fear of infecting others (especially loved ones who could have poor outcomes) (27). In health care settings, the need for isolation in response to a false-positive result can lead to staff shortages, adding further stress on other employees and patients. False-positive APOCT results in acute care settings have resulted in unnecessary cancellation or postponement of treatment/procedures and also risk an individual possibly being exposed to SARS-CoV-2 if moved into a COVID-19 treatment unit based on the APOCT result (28). Thus, we strongly recommend proper education about the meaning of APOCT results and ensuring processes are in place for timely confirmation with rRT-PCR if the APOCT is implemented.

The setup of asymptomatic testing programs using APOCTs requires considerable input of resources and logistical organization. To ensure the utmost quality of testing, APOCTs should undergo appropriate test verification and creation of standard operating procedures and training programs, and they should include appropriate medical and operational oversight through an accredited laboratory when implementing the associated POCT program (29). Information technology specialists are generally required to establish reporting systems so that results can be accurately captured to facilitate public health reporting, disease notification, and contact tracing. Furthermore, ongoing oversight to address reporting errors, test distribution, and ongoing test validation must be built into the APOCT infrastructure (30). The impact of false-positive results, alongside these important operational considerations, must be accounted for when deciding on the benefit of an APOCT screening program.

The principal limitation of this study is that only positive APOCTs were confirmed by rRT-PCR, and thus no other test parameters other than a PPV could be calculated. The data, however, still confirm, that in a large asymptomatic HCW population, APOCTs have a low PPV. The major strengths of this study lie in the large number of tests performed, participating sites, and inclusion of two different APOCT platforms.

The utility of screening asymptomatic individuals with a COVID-19 APOCT is best determined by weighing the costs and benefits specific to each setting. With widespread vaccination increasing and disease prevalence falling, fewer positive test results are likely to be found among asymptomatic persons in the coming months (with a large proportion of them being false positives), leading such endeavors to be quite costly from both materials and human resources perspectives for most health care systems. Therefore, it is important to reconsider the value and effectiveness of asymptomatic APOCT COVID-19 programs over the long term, especially in the context of increasing population immunity.
